# Incomplete Pattern of Steroidogenic Protein Expression in Functioning Adrenocortical Carcinomas

**DOI:** 10.3390/biomedicines8080256

**Published:** 2020-07-30

**Authors:** Sofia S. Pereira, Madalena M. Costa, Celso E. Gomez-Sanchez, Mariana P. Monteiro, Duarte Pignatelli

**Affiliations:** 1 Instituto de Investigação e Inovação em Saúde (I3S), Universidade do Porto, 4200-135 Porto, Portugal; spereira.bq@gmail.com; 2 Cancer Signalling & Metabolism group, Institute of Molecular Pathology and Immunology of the University of Porto (IPATIMUP), 4200-135 Porto, Portugal; 3 Endocrine, Cardiovascular & Metabolic Research group, Multidisciplinary Unit for Biomedical Research (UMIB), University of Porto, 4050-313 Porto, Portugal; mcosta@icbas.up.pt (M.M.C.); mpmonteiro@icbas.up.pt (M.P.M.); 4 Department of Anatomy, Instituto de Ciências Biomédicas Abel Salazar (ICBAS), University of Porto, 4050-313 Porto, Portugal; 5 Endocrinology Section, G.V. (Sonny) Montgomery VA Medical Center, Jackson, MS 39216, USA; cgomez-sanchez@umc.edu; 6 Department of Pharmacology and Toxicology, University of Mississippi Medical Center, Jackson, MS 39216, USA; 7 Department of Endocrinology, Centro Hospitalar Universitário de São João, 4200-319 Porto, Portugal; 8 Department of Biomedicine, Faculty of Medicine of the University of Porto, 4200-319 Porto, Portugal

**Keywords:** adrenocortical tumors, adrenocortical carcinomas, steroidogenic enzymes, differential diagnosis, 11β-hydroxylase

## Abstract

Autonomous steroid secretion is a common feature of adrenocortical carcinomas (ACC), although not always clinically evident owing to inefficient steroidogenesis with increased release of steroid precursors. Our study aim was to analyze the expression profile of four key proteins involved in the steroidogenesis cascade, in different adrenocortical tumors. Expression of proteins involved in steroidogenesis, namely steroidogenic acute regulatory protein (StAR), 11β-hydroxylase (CYP11B1), aldosterone synthase (CYP11B2) and 17α-hydroxylase (CYP17A1), were analyzed by immunohistochemistry in ACC (*n* = 14), adenomas presenting with Cushing’s syndrome (ACAc) (*n* = 11) and clinically non-functioning adenomas (ACAn) (*n* = 15). A percentage of the stained area for each protein was analyzed using ImageJ software for computerized morphometric quantification. CYP11B1, StAR and CYP17A1 expression were significantly lower in ACC when compared to ACAc. In addition, ACC presented co-staining cells for CYP11B1 and CYP11B2. CYP11B1 was the steroidogenic enzyme with the most discriminative power to distinguish ACC from ACAc, with a sensitivity of 100%, specificity of 92%, and an expression higher than 4.44%, indicating the presence of a cortisol secreting adenoma. ACC depicts an incomplete pattern of steroidogenic protein expression, with decreased CYP11B1 and CYP17A1, which could explain the predominant secretion of steroid precursors.

## 1. Introduction

Adrenocortical carcinomas (ACC) are rare tumors and are largely associated with a poor prognosis [1]. The majority of ACC autonomously produce steroids and present as clinically functioning tumors in 40–60% of cases, of which 50–80% of patients exhibit Cushing’s syndrome [2,3,4]. In addition, despite being capable of steroid production, some ACC do present without a clinically apparent hormonal syndrome.

Previous steroid metabolomics studies using gas chromatography/mass spectrometry (GS–MS) demonstrated that the majority of patients with ACC have higher androgen and glucocorticoid urinary levels when compared to patients with adrenocortical adenomas (ACA). In addition, the authors found that ACCs secrete and release predominantly intermediate metabolites, which provides an explanation for the absence of clinically apparent hormonal overproduction syndromes [5,6,7,8,9].

In addition to the knowledge contribution on ACC biology, urinary steroid metabolomics profile analysis of patients harboring ACC and ACA also provided strong evidence that some steroid metabolites, such as tetrahydro-11-deoxycortisol (THS) and pregnanediol, are promising biomarkers for the differential diagnosis of adrenocortical tumors (ACT) [8]. These findings are clinically relevant since ACC diagnosis remains challenging and lacks diagnostic accuracy, in particular for tumors with borderline clinical, histological, and radiological characteristics [2,10].

**Figure 1:**
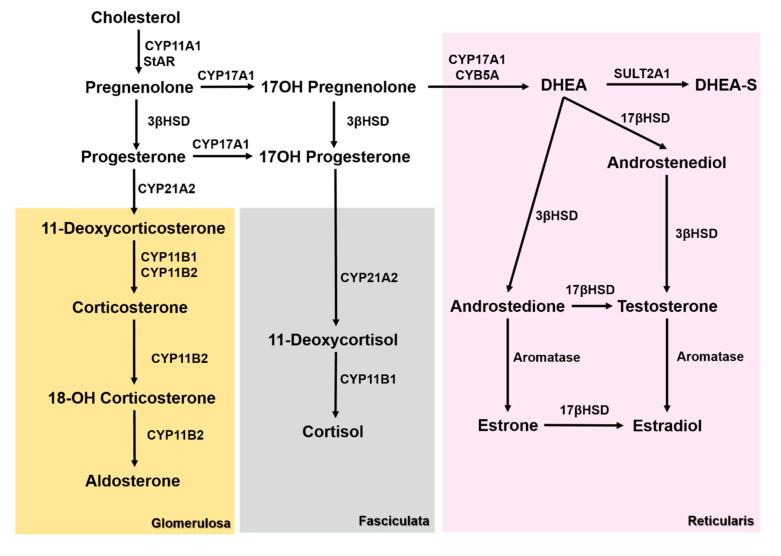
Steroidogenesis pathways in the different adrenal cortex layers. 3βHSD—3β-hydroxysteroid dehydrogenase, 17βHSD—17β-hydroxysteroid dehydrogenase, CYB5A—Cytochrome B5A, CYP11A1—cholesterol side chain cleavage enzyme, CYP11B1—11β-hydroxylase, CYP11B2—aldosterone synthase, CYP17A1—17α-Hydroxylase, CYP21A2—21α-hydroxylase, dehydroepiandrosterone (DHEA), dehydroepiandrosterone sulfate (DHEA-S), StAR—steroidogenic acute regulatory protein, and SULT2A1—Sulfotransferase 2A1.

The reasons behind the observed ACC steroid secretion pattern remains to be elucidated, but it may be attributed to the undifferentiated status of the tumor cells expressing an incomplete pattern of enzymes involved in the steroidogenic cascade (Figure 1).

The aim of this study was to analyze the expression of four key proteins involved in adrenal steroidogenesis, namely steroidogenic acute regulatory protein (StAR), 11β-hydroxylase (CYP11B1), aldosterone synthase (CYP11B2), and 17α-hydroxylase (CYP17A1), in different adrenocortical tumors in order to understand the potential utility of these molecular markers for diagnosis in a clinical setting.

## 2. Experimental Section

### 2.1. Case Selection

Adrenal tissue was obtained during elective surgical procedures from patients with adrenal ACT (*n* = 40), comprising ACC (*n* = 14) and ACA (*n* = 26), including non-functioning ACA (ACAn) (*n* = 15) and adenomas with clinical features of Cushing’s syndrome (ACAc) (*n* = 11). Malignancy was based on the Weiss score. Adrenocortical tumors with Weiss score >4 were classified as carcinomas, and those with a Weiss score <2 were classified as adenomas. Functionality was assessed through dexamethasone tests and 24h urine-free cortisol. Half of the ACCs were functioning tumors.

The patients provided a written informed consent accepting that a tumor sample would be stored in the tumor bank of the Department of Pathologic Anatomy—Centro Hospitalar de São João, to posteriorly be used in research studies.

The study was conducted in accordance with the Declaration of Helsinki, and the protocol was approved by the Ethics Committee of Centro Hospitalar Universitário de São João (Porto, Portugal) (Ethical approval code: CE 62-14, approved on 10 December 2015).

### 2.2. Immunohistochemistry and Analysis

The CYP11B1, CYP11B2, and CYP17A1 antibodies used in this study were developed by Professor Celso E. Gomez-Sanchez from the Medical Center, University of Mississippi, USA, and the immunohistochemistry (IHC) protocol was performed as previously described [11]. Briefly, 3 μm formalin-fixed paraffin-embedded tissue sections mounted on adhesive microscope slides (StarFrost, Knittel Glass, Braunschweig, Germany) were deparaffinized and rehydrated in graded alcohols, then they underwent 45 min heating in a temperature-controlled water bath (99.9 °C) with ethylenediaminetetraacetic acid (EDTA) (E5134, Sigma-Aldrich, St Louis, MO, USA) solution 1 mM at pH 9 with sodium dodecyl sulfate (SDS) 0.05%, for antigen retrieval. Endogenous peroxidase inhibition was performed using hydrochloric acid at 0.02 N for 20 min before overnight incubation with the primary antibodies: CYP11B1 (1:100), CYP11B2 (1:500) or CYP17A1 (1:500) at 4 °C. Detection of the immune reaction was performed by incubation for 60 min with the commercial Dako REAL™ EnVision™ Detection System (ref.: K5007, Dako, Glostrup, Denmark), which includes a dextran backbone with peroxidase (HRP) molecules coupled to goat secondary antibody molecules against rabbit immunoglobulins. DAB (3,3′-diaminobenzidine, included in the same commercial Dako Kit) was used as a chromogen, and Mayer’s hematoxylin (ref.: HX390929, Merck, Darmstadt, Germany) was used for nuclear counterstaining.

For StAR, antigen recuperation was performed by boiling in a pressure cooker for 3 min in a 0.01 M citrate buffer at pH 6.0 with 0.05% Tween 20. The endogenous peroxidase was blocked with 3% hydrogen peroxide in methanol, followed by normal serum pre-treatment for 30 min and overnight incubation at 4 °C, with the primary rabbit anti-human polyclonal antibodies against StAR (HPA023644; 1:100; Atlas Antibodies). Slides were then incubated with the secondary antibodies at a 1:200 dilution (Polyclonal swine anti-rabbit, Dako, Glostrup, Denmark), followed by avidin-biotin peroxidase complexes (1:100, Vector Laboratories, Inc., Burlingame, CA, United States) for 30 min. Diaminobenzidine (K3468, Dako, Glostrup, Denmark) was used as a chromogen, and hematoxylin was used for nuclear counterstaining. Normal adrenal tissue was used as a positive control, and omission of primary antibody incubation was used as a negative control.

Each immunohistochemistry stained slide was scanned using the Olympus VS110 virtual slide scanning system, and the images were acquired through VS-ASW software (version 2.3 for Windows, Olympus, Tokyo, Japan). Each image was then analyzed using FIJI software (National Institutes of Health, USA) and its color deconvolution plugin (HDab), which allows isolation of the stained area from the initial image.

Using a similar protocol, the total tissue area was also calculated. The percentage of the stained area for each protein was assessed by calculating the ratio between the stained area and the total tissue area, as previously described [12,13].

### 2.3. Immunofluorescence

For antigen retrieval, slides underwent heating by microwaving at 900 W in 10 mM citrate buffer (pH 6.0) with 0.05% Tween 20 for 15 min. Autofluorescence inhibition was performed by incubation with Sudan black B 0.5% in 70% of alcohol for 30 min. Afterwards, slices were incubated overnight with both primary antibodies anti-CYP11B1 (1:30) and anti-CYP11B2 (1:200) at 4 °C. Slides were then incubated for 2 h with a cocktail containing a fluorescent secondary antibody goat anti-rat (1:200, #4417, Cell Signaling Technology, Danvers, MA, USA) and goat anti-mouse (1:750, #4408S, Cell Signaling Technology, Danvers, MA, United States). The slides were mounted and counterstained with DAPI hard set (ref. H1500, Vector Laboratories, Burlingame, CA, United States).

### 2.4. Statistical Analysis

All statistical analyses were performed using GraphPad Prism (version 7.00 for Windows, San Diego, CA, United States). The percentage of the stained area for each marker is represented as mean ± standard error of the mean (SEM). Normality for continuous variables was assessed using the D’Agostinho and Pearson test. For normally distributed variables that passed this test, a one-way ANOVA test with post-hoc Tukey was used to compare the means of the three study groups. The continuous variables that did not follow a normal distribution were analyzed using the Kruskal–Wallis test with a post-hoc Dunn’s test. Differences between the groups were considered statistically significant when *p* < 0.05.

The area under the receiver operating characteristic (ROC) curve was used to determine diagnostic accuracy of the markers with significant results. Based on the area under the curve (AUC), the test was considered excellent for values ranging between 0.90 and 1.00, good when between 0.80 and 0.90, fair if between 0.70 and 0.80, poor for values between 0.60 and 0.70, and failed if below 0.60 [14]. The analysis of ROC curves also allowed us to obtain the best cut-off values for the differential diagnosis.

## 3. Results

### 3.1. CYP11B1 Expression in ACC Is Low

**Figure 2:**
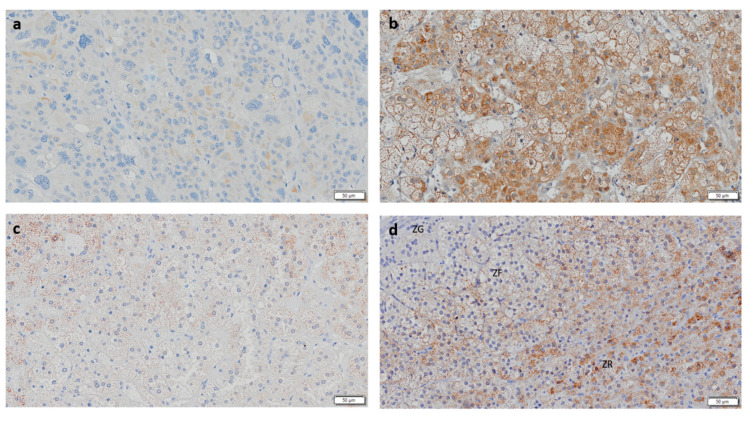
Immunohistochemistry staining for CYP11B1 (Scale = 50 µm). (**a**) Adrenocortical carcinoma, (**b**) adrenocortical adenoma with Cushing’s syndrome, (**c**) non-functioning adrenocortical adenoma, and (**d**) normal adrenal gland. ZG—zona glomerulosa; ZF—zona fasciculata; ZR—Zona reticularis.

**Figure 3:**
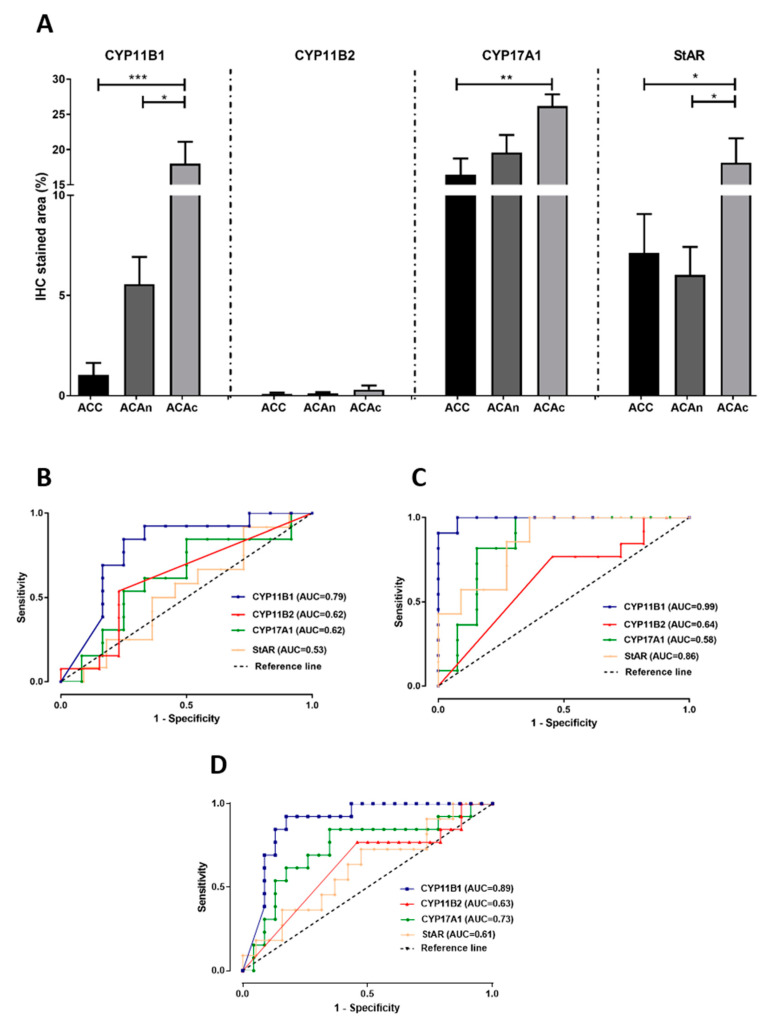
Graphical representation of the percentage of CYP11B1, CYP11B2, CYP17A1 and StAR expression in the studied groups (**A**). ROC curves to distinguish adrenocortical carcinomas (ACC) from non-functioning adrenocortical adenomas (ACAn) (**B**); ACC from adenomas with Cushing’s syndrome (ACAc) (**C**); and ACC from total adenomas (**D**) with the respective area under the curve (AUC). * *p* < 0.05; ** *p* < 0.01; *** *p* < 0.001; dashed lines represent the reference lines of the ROC curves.

CYP11B1 expression was present in all ACAc and in the majority of ACAn (*n* = 13/15, 86.7%) and ACC (*n* = 9/14, 64.3%) (Table 1 and Figure 2). Staining for CYP11B1 was significantly higher in ACAc (17.99 ± 13.12%) when compared to ACAn (5.56 ± 1.35%, *p* < 0.05) and ACC (1.04 ± 0.59%, *p* < 0.001) (Figure 3A).

ROC curve analysis showed that CYP11B1 is a marker with high accuracy for differential diagnosis between ACC and ACAc, with an AUC of 0.99 (Figure 3C) and good accuracy for differential diagnosis between ACC and total ACA (Figure 3D).

### 3.2. CYP11B2 and CYP11B1 Dual Negativity Is Highly Suggestive of Malignant Adrenocortical Tumors

**Figure 4:**
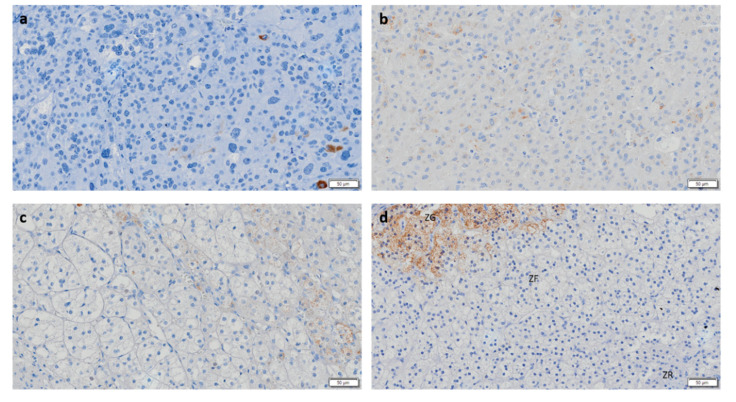
Immunohistochemistry staining for CYP11B2 (Scale = 50 µm). (**a**) Adrenocortical carcinoma, (**b**) adrenocortical adenoma with Cushing’s syndrome, (**c**) non-functioning adrenocortical adenoma, and (**d**) normal adrenal gland. ZG—zona glomerulosa; ZF—zona fasciculata; ZR—Zona reticularis.

CYP11B2 IHC staining was negative in the majority of ACC, and in the rare tumors with positive staining, expression was found to be low (Figure 4a). After ROC curve analysis, CYP11B2 exhibited insufficient accuracy for differential diagnosis of ACT given the low AUC values observed (Figure 3B,C). However, CYP11B1 and CYP11B2 dual negativity is highly suggestive of ACC, since it was only observed in ACC (28.6%) and in 1 ACAn (6.7%). This pattern has a specificity of 100 or 96% for malignancy in functioning or non-functioning ACT, respectively.

### 3.3. CYP17A1 Expression in ACC Is Low

**Figure 5:**
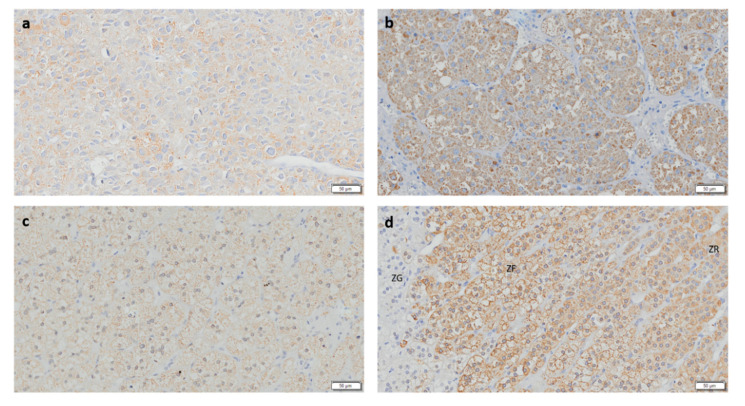
Immunohistochemistry staining for 17α-hydroxylase (CYP17A1) (Scale = 50 µm). (**a**) Adrenocortical carcinoma, (**b**) adrenocortical adenoma with Cushing’s Syndrome, (**c**) non-functioning adrenocortical adenoma, and (**d**) normal adrenal gland. ZG—zona glomerulosa; ZF—zona fasciculata; ZR—Zona reticularis.

CYP17A1 expression was positive in every ACC and ACAc, as well as in the majority of ACAn (*n* = 14/15, 93.3%) (Table 1). CYP17A1 expression was significantly lower in ACC (16.44 ± 2.29) when compared with ACAc (26.19 ± 1.63, *p* < 0.01) (Figure 3 and Figure 5). However, ROC curves depicted low AUC, indicating that this enzyme has poor accuracy in differential diagnosis (Figure 3).

### 3.4. StAR Expression Is Decreased in ACC and ACAn

**Figure 6:**
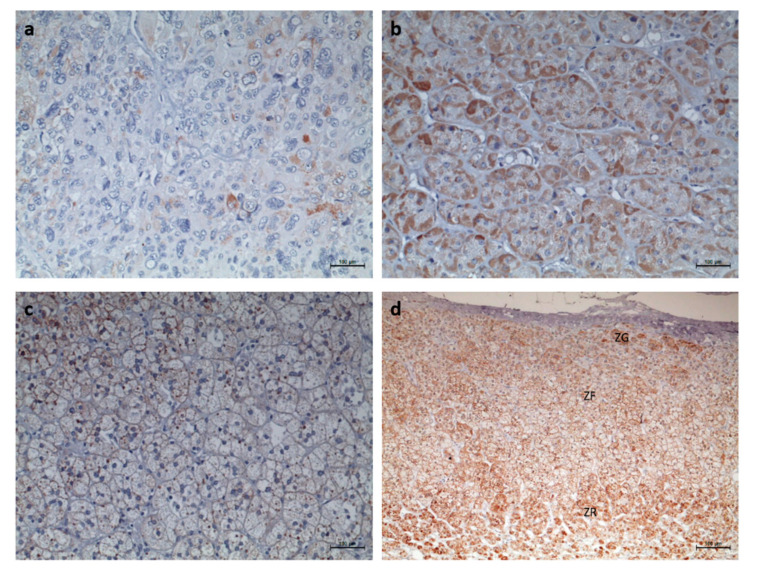
Immunohistochemistry staining for StAR (Scale = 100 µm). (**a**) Adrenocortical carcinoma, (**b**) adrenocortical adenoma with Cushing’s syndrome, (**c**) non-functioning adrenocortical adenoma, and (**d**) normal adrenal gland. ZG—zona glomerulosa; ZF—zona fasciculata; ZR—Zona reticularis.

Positivity for the StAR antibody was present in every ACT (Figure 6 and Table 1), although significant differences in the stained area were only found between ACAc (18.12 ± 1.40%) and ACC (7.11 ± 1.95%, *p* < 0.05). StAR expression was significantly lower in ACAn (6.02 ± 1.40%) than in ACAc (18.12 ± 1.40%, *p* < 0.05) and similar to ACC (7.11 ± 1.95%) (Figure 3A). The ROC curve analysis to access the accuracy of StAR for differential diagnosis between ACA and ACC has a yield AUC of 0.86, suggesting that StAR is a good molecular marker to distinguish ACC from ACAc, but not between ACC and ACAn (Figure 3B,C).

### 3.5. ACCs Present Cells That Express Both CYP11B1 and CYP11B2

**Figure 7:**
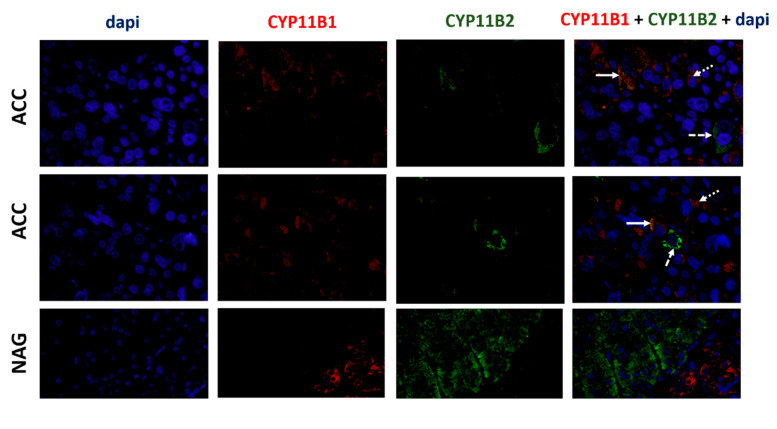
Immunofluorescence staining for CYP11B1 (red), CYP11B1 (green), and dapi (blue) in adrenocortical carcinomas (ACC) and normal adrenal gland (NAG) (400×). Solid arrows indicate cells co-staining for CYP11B1 and CYP11B2, dotted arrows indicate examples of cells only staining for CYP11B1, and dashed arrows indicate examples of cells only staining for CYP11B2, in ACC.

Immunofluorescence for CYP11B1 and CYP11B2 was performed only in the cases that expressed CYP11B1 and CYP11B2 by IHC. Normal adrenal glands were used as positive controls. We found that only ACC presented co-staining cells for both steroidogenic enzymes. Those cells were distributed throughout the tumors. However, the majority of cells only stained for CYP11B1 and CYP11B2 individually (Figure 7).

## 4. Discussion

Despite the fact that the majority of ACCs are capable of autonomous steroid production, these tumors do not always present as clinically functioning with hormone secretion syndromes [15]. Steroidogenesis in ACC is known to be dysfunctional, and the use of steroid metabolite levels as a tool for diagnosis was previously suggested, despite the utility of serum steroid routine analysis being highly controversial [5,6,7,8,9]. Arlt et al. performed steroid metabolomics by gas chromatography/mass spectrometry in the urine of ACT patients and observed that a combined androgen and glucocorticoid excess was present in 69% of the analyzed ACC, while routine biochemistry analysis only identified 27% of the cases [6]. Eighty-five percent of the ACC patients presented with an accumulation of steroid precursors rather than mature steroids, suggesting incomplete steroidogenesis [6]. This hormonal profile could be due to an incomplete pattern of steroidogenic enzyme expression, which warranted characterization. Thus, our aim was to analyze the expression profile of four key proteins involved in the steroidogenesis cascade, namely StAR protein, CYP11B1, CYP11B2 and CYP17A1, in different types of adrenocortical tumors.

Our results have shown that ACCs have a decreased expression of CYP11B1, StAR and CYP17A1 when compared to ACAc, while CYP11B1 is the steroidogenic enzyme with the highest discriminative power to distinguish ACC from ACAc with a sensitivity and specificity of 100 and 92%, respectively, for a cut-off value of 4.44%. Besides that, 28.6% of ACCs were negative for both CYP11B1 and CYP11B2, while this pattern was only found in one ACAn and in none of the ACAc. Although isolated CYP11B2 negativity was not useful for diagnosis, CYP11B1 and CYP11B2 dual negativity is absolutely suggestive of ACC, with a specificity of 100 or 96% in functioning and non-functioning ACT, respectively. When comparing the two subgroups of benign tumors, CYP11B1 and StAR were the steroidogenic proteins that were most differentially expressed in ACAn and ACAc, with lower levels of both proteins found in non-functioning ACA.

Previous studies that focused on analysis of the urine steroid metabolomics profiles of patients harboring ACC and ACA found that THS, a metabolite of 11-deoxycortisol, was the steroid with the highest discriminative power to differentiate ACA from ACC [5,6,7,8,9]. The abundance of THS suggests that CYP11B1, the enzyme responsible for the conversion of 11-deoxycortisol into cortisol, is either dysfunctional or exhibits decreased expression, as was observed in the present study. The progesterone metabolite pregnanediol was also higher in the urine of ACC patients [8]. This finding corroborates our own observation of decreased expression of CYP17A1 in ACC, since CYP17A1 is responsible for conversion of progesterone into 17α-hydroxypregnenolone.

Few studies have analyzed the expression profile of the steroidogenic enzymes in ACC. Sasano et al. described that ACC presented a disorganized pattern of steroidogenic enzyme expression, while non-functioning ACCs were devoid of CYP11B1 and CYP17A1 and depicted lower levels of expression of other enzymes (21-hydroxylase, 3-β-hydroxysteroid dehydrogenase and CYP11A1) as compared to functioning ACC presenting with Cushing’s syndrome [16]. Uchida et al. reported a case of an ACC presenting with mild primary aldosteronism and subclinical Cushing’s syndrome, in which CYP11B1, CYP11B2 and 3-β-hydroxysteroid dehydrogenase were poorly expressed as detected by immunohistochemistry and varied across different areas of the tumor. Moreover, some CYP11B2-positive cells also expressed the CYP17A1 enzyme, a feature that does not occur in normal adrenal cells [17]. In accordance with these results, we found that ACC presented cells that expressed both CYP11B1 and CYP11B2, reinforcing the fact that the normal coordinated expression of enzymes involved in steroidogenesis is disturbed in ACC.

Although this study presents some new and important data about adrenocortical tumor biology, some limitations need to be acknowledged. First, our results were based on a limited number of tumors, and steroid precursors were not assessed in these patients. In addition, although CYP11B2 and CYP11B1 dual negativity has a high specificity to identify malignant ACT, the sensitivity is low.

In addition, although our study and most other steroid metabolomics studies support the fact that the majority of ACCs present an altered steroidogenesis with low levels of key steroidogenic enzymes, we must point out that other studies reported a great variation in the ACC steroidogenic activity and suggested that this may be dependent on tumor size, with a trend for small tumors to have higher CYP11B1 activities [18]. In addition, the NCI-H295 cell line is derived from an ACC and expresses most of the key enzymes necessary for steroidogenesis [19], so our results need to be validated in a prospective study with a larger ACT patient cohort.

In conclusion, our study provides significant evidence that CYP11B1, StAR, and CYP17A1 expression is lower in ACC when compared to benign ACAc. The incomplete pattern of steroidogenic enzyme expression could justify the increased secretion of steroid metabolite precursors witnessed in ACC. CYP11B1 was shown to be a highly accurate molecular marker for differential diagnosis between ACC and ACAc. In addition, CYP11B1 and CYP11B2 dual negativity was shown to be very specific for malignancy.

## Figures and Tables

**Table 1:** Frequency of CYP11B1, CYP11B2, CYP17A1 and StAR immunostaining positivity in the different adrenocortical tumors.

Adrenocortical Tumors	Steroidogenic Proteins	Negative	Positive
ACC (*n* = 14)	CYP11B1	5 (35.7%)	9 (64.3%)
	CYP11B2	11 (78.6%)	3 (21.4%)
	CYP17A1	0 (0.00%)	14 (100.0%)
	StAR	0 (0.00%)	14 (100.0%)
ACAc (*n* = 11)	CYP11B1	0 (0.00%)	11 (100.0%)
	CYP11B2	5 (45.5%)	6 (54.5%)
	CYP17A1	0 (0.00%)	11 (100.0%)
	StAR	0 (0.00%)	11 (100.0%)
ACAn (*n* = 15)	CYP11B1	2 (13.3%)	13 (86.7%)
	CYP11B2	7 (46.7%)	8 (53.3%)
	CYP17A1	1 (6.7%)	14 (93.3%)
	StAR	0 (0.00%)	15 (100.0%)

ACC—Adrenocortical carcinomas, ACAc—Adrenocortical adenomas with Cushing’s syndrome, ACAn—non-functioning adrenocortical adenomas, CYP11B1—11β-hydroxylase, CYP11B2—Aldosterone synthase, CYP17A1—17α-hydroxylase, and StAR—Steroidogenic acute regulatory protein.
